# Unilateral Acroangiodermatitis: From Histopathologic Confirmation to Treatment with PDL

**DOI:** 10.3390/dermatopathology12040035

**Published:** 2025-10-08

**Authors:** André Aparício Martins, José Carlos Cardoso, André Pinho

**Affiliations:** Dermatology and Venereology Department, Unidade Local de Saúde de Coimbra, 3004-561 Coimbra, Portugal

**Keywords:** acroangiodermatitis, Kaposi sarcoma, pulsed dyed laser

## Abstract

Acroangiodermatitis is an uncommon angioproliferative dermatosis, related to chronic circulatory diseases, such as chronic venous insufficiency and arteriovenous malformations. We describe the case of a 32-year-old healthy male presenting with a pruritic, brownish lesion on the dorsal surface of the left foot, evolving for ten years. Physical examination revealed a brown plaque, with a verrucous surface, on the distal dorsum and medial border of the left foot. Histopathology disclosed a marked neovascularization of the upper dermis, associated with erythrocyte extravasation and hemosiderin deposition. Immunochemistry for HHV-8 was negative. CT angiography revealed multiple serpiginous vessels on the dorsum of the left foot, suggestive of a venous malformation. The diagnosis of acroangiodermatitis was established and the patient started topical corticosteroids and compression stockings, without improvement. Although scarcely described in the literature, treatment with PDL was proposed given the vascular proliferation confined to the papillary dermis. After two sessions, a significant improvement was observed. This case emphasises dermatopathology as the gold standard for the differential diagnosis with Kaposi sarcoma. In addition, it highlights PDL as a promising therapeutic option, based on the superficial histopathological location.

## 1. Introduction

Acroangiodermatitis (AAD) or pseudo-Kaposi sarcoma is an underreported reactive angioproliferative dermatosis that frequently involves the lower extremities [1,2]. Its aetiopathogenesis is based on a reactive proliferation of small blood vessels due to a chronic circulatory disturbance [3]. Endothelial proliferation, mediated by vascular endothelial growth factor (VEGF), may be triggered by distal hypoxia [4]. This stimulus may occur in various vascular conditions, such as chronic venous insufficiency, congenital arteriovenous malformations and acquired iatrogenic arteriovenous fistulas (for haemodialysis) [2,4]. Occasionally, AAD is associated with amputation stumps (suction socket prosthesis), paralysed limbs and thromboembolic events due to thrombophilia [5]. Although benign, AAD clinically resembles Kaposi sarcoma (KS). Thus, differential diagnosis is mandatory, with dermatopathology playing an essential role.

## 2. Case Description

**Figure 1:**
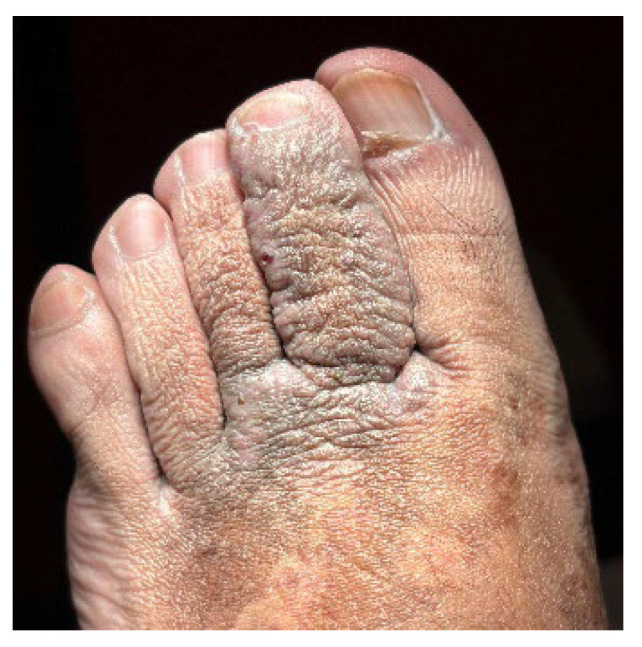
Brown plaque, with a focal erythematous hue, on the distal dorsum and medial border of the left foot, with a verrucous surface in the 2nd, 3rd, and 4th toes.

**Figure 2:**
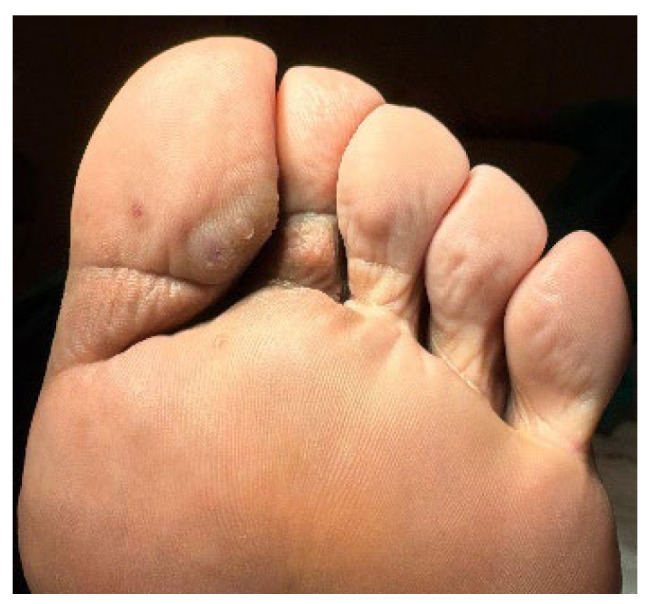
Lesion extension to the plantar surface of the distal and proximal/middle phalanges of the 1st and 2nd toes, respectively.

A 32-year-old healthy male presented with a brownish lesion on the dorsal surface of the left foot, evolving for 10 years. During this time the lesion progressively increased in size, involving the second, third, and fourth toes, and the medial border of the foot. Initially, the patient referred mild local pain, but in recent months developed mild pruritus. He denied systemic symptoms, mucosal involvement and similar lesions in other anatomical regions. He had no personal history of venous insufficiency, cardiovascular diseases or thromboembolic events. Physical examination revealed a brown plaque, with a focal erythematous hue, irregular borders, and ill-defined margins on the distal dorsum and medial border of the left foot. The lesion extended to the dorsal surface of the second, third, and fourth toes, here presenting with a thicker verrucous surface (Figure 1). Additionally, it extended towards the plantar surface of the distal and proximal/middle phalanges of the first and second toes, respectively (Figure 2). There were no palpable thrill or bruit and no oedema or varicous veins.

The differential diagnosis included AAD, KS, and verrucous lichen planus. Complete blood count, serum electrolytes, and renal and hepatic function were normal. Serologies for HIV and HCV were negative and showed immunity for HBV. Duplex ultrasonography of the left lower limb was normal. There was no valvular insufficiency, no dilatated perforator veins, and no evidence of deep and superficial venous thrombosis. Arterial vessels did not demonstrate any atheromatous plaques and the spectral Doppler revealed a normal triphasic flow pattern. However, the CT angiography revealed multiple serpiginous vessels on the ankle and dorsum of the left foot, suggestive of a venous malformation. The arterial axis was patent, with no evidence of calcified atherosclerosis.

**Figure 3:**
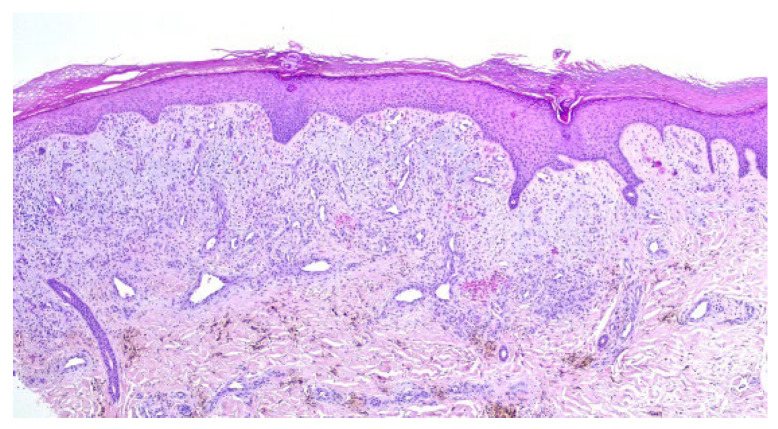
Moderate acanthosis with focal spongiosis, associated with a marked neovascularization in the upper dermis, erythrocyte extravasation, and hemosiderin deposition.

**Figure 4:**
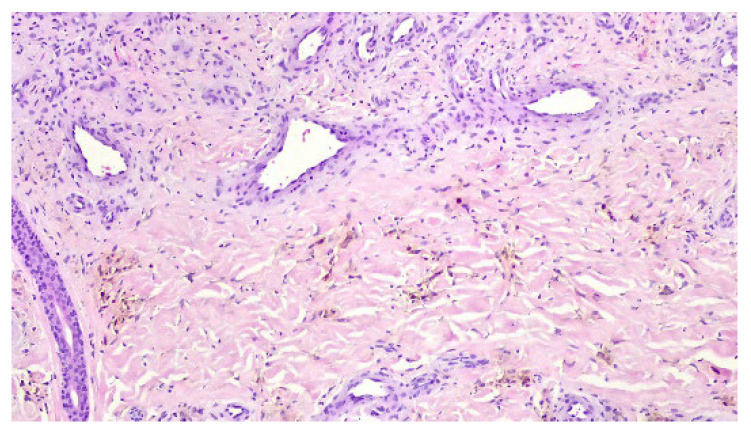
Lobules of thick-walled capillaries and hemosiderin deposition.

**Figure 5:**
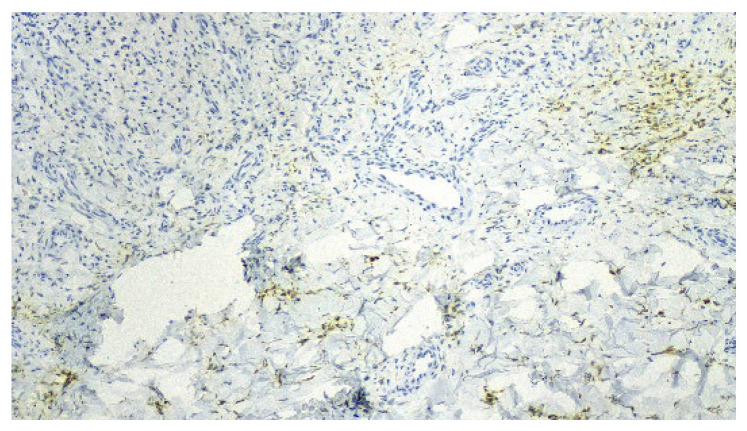
Negative immunochemistry for HHV-8.

A punch biopsy was performed, and the histopathology disclosed moderate acanthosis, with focal spongiosis and small lymphocyte exocytosis. In the upper dermis, a marked neovascularization, composed of lobules of thick-walled capillaries, was observed in association with erythrocyte extravasation and hemosiderin deposition (Figure 3 and Figure 4). Immunochemistry for HHV-8 was negative (Figure 5). The clinicopathological correlation led to the diagnosis of AAD due to a venous malformation. This case illustrates a possible pathophysiological overlap with the venous malformation contributing to venous insufficiency.

**Figure 6:**
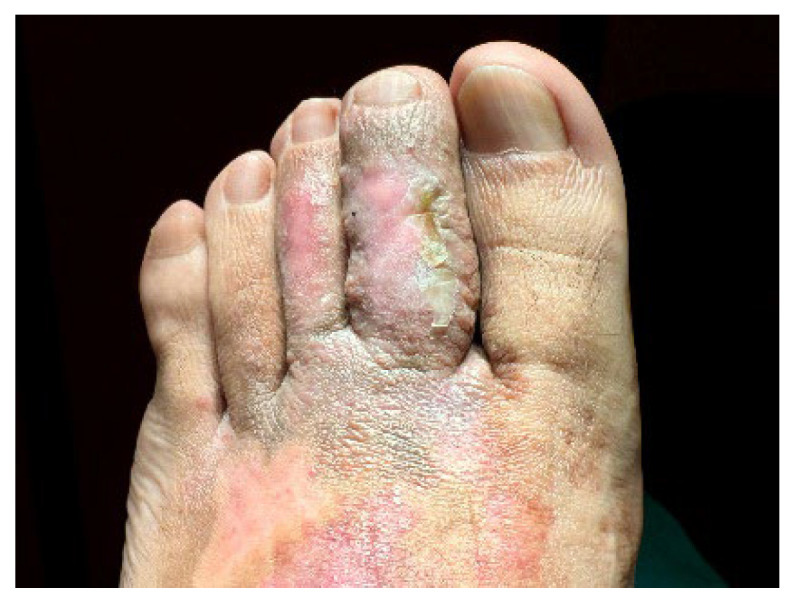
Dorsal aspect of the patient’s lesion following two PDL sessions.

**Figure 7:**
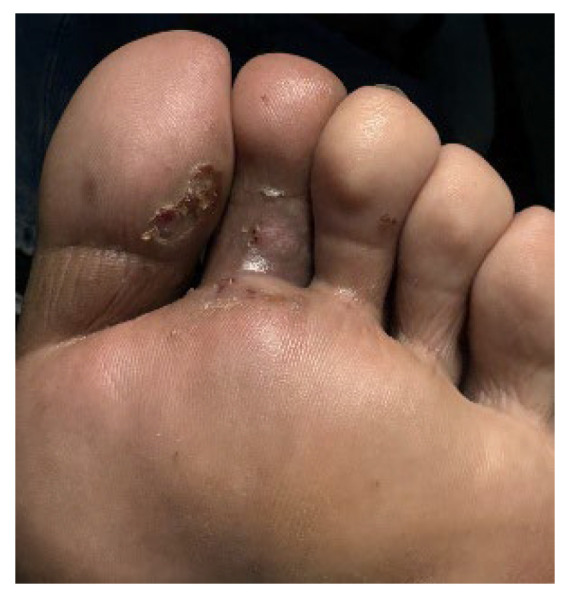
Plantar aspect of the patient’s lesion following two PDL sessions.

Initially, grade III compression stockings and daily diflucortolone valerate 1 mg/g cream was advised for 6 months. Despite pruritus improvement, the physical examination revealed no changes and pulsed-dye laser (PDL) was proposed. The procedure was performed under digital nerve block, using a Synchro VasQ^®^ laser (Deka^®^, Calenzano (FI), Italy) with a fluence of 7–10 J/cm^2^, pulse duration of 0.5–3 ms, and a spot size of 10–12 mm. After two sessions, a significant improvement was noticed. The whole lesion became thinner, and some areas acquired an erythematous and whitish hue, consistent with post-inflammatory changes (Figure 6 and Figure 7). The patient referred only local pain during the first days after PDL sessions, without other adverse events. After recovery, he quickly resumed daily life activities. Currently, the patient continues to use grade III compression stockings and was referred to vascular surgery. The need for additional laser sessions will depend on the patient’s clinical course.

## 3. Discussion

AAD is a reactive proliferation of small blood vessels in response to a chronic circulatory disturbance [3]. Chronic venous insufficiency, congenital arteriovenous malformations (e.g., Klippel–Trenaunay and Prader–Labhart–Willi syndromes) and acquired iatrogenic arteriovenous fistulas (for haemodialysis) are frequently reported causes [5,6]. Other aetiologies have been described, such as the use of poorly fitting suction-type devices in amputation stumps, paralysed limbs (including congenital myopathies), and thromboembolic events due to thrombophilia (20210A prothrombin mutation and activated protein C resistance) [5,6]. Although poorly understood, AAD physiopathology may involve VEGF, PGE1, or heparin like factor, which may contribute to endothelial proliferation in distal hypoxic areas [4,7]. A possible role for mast cells and microtrauma has also been described [4,7]. In this context, several pathophysiological hypotheses have been proposed, including the following: the angiogenic response (fibroblast proliferation and endothelial hyperplasia) to high perfusion rates; the increased venous pressure that may stimulate endothelial proliferation; and the distal ischemia that may stimulate local secretion of VEGF, leading to endothelial proliferation [8].

AAD usually presents as purple or reddish-brownish papules, plaques, or nodules on the lower legs and dorsal feet/toes [6,8]. Lesions may be painful and tend to evolve slowly [2,3]. Ulcers and verrucous plaques, as reported in our case, have also been described [4,6]. In chronic venous insufficiency the lesions are usually bilateral, while in other vascular or neurological diseases tend to be unilateral [5]. Although rare, upper limb involvement is described in association with arteriovenous fistulas for haemodialysis [3].

AAD is subdivided in two variants: Mali type and Stewart–Bluefarb type. Mali type is the most common variant and is classically associated with severe chronic venous insufficiency of the lower limbs [4]. In this subtype, the chronic oedema reduces perfusion and triggers local neovascularization [2,6]. It frequently affects elderly patients and is bilateral in presentation [2]. Stewart–Bluefarb type is associated with arteriovenous malformations/fistulae, amputees, and hemiplegic patients [2]. Here, the high perfusion rate triggers an angiogenic response [2,6]. This variant is more common in younger patients, with a unilateral presentation, as described in our case. Imaging techniques contribute to distinguish the two variants of AAD [2,4]. Duplex ultrasonography is the first line, but the suspicious findings must be confirmed by CT or MRI angiography [2].

Although benign, AAD may be associated with the local and systemic complications of an underlying arteriovenous malformation. Refractory pain, physical impairment, haemorrhage, recurrent infection, skin ulceration, and congestive heart failure are described [2].

The main differential diagnosis of AAD is KS. Lichen planus, stasis dermatitis, pigmented purpura, lichen aureus, lichen simplex chronicus, basal cell carcinoma, hemangioma, lymphangioma, and lymphangiosarcoma are other differential diagnoses [3]. In verrucous presentations, deep mycosis, squamous cell carcinoma, and angiosarcoma should also be considered [6]. Angiopericytomatosis, intravascular histiocytosis, diffuse dermal angiomatosis, and reactive or glomeruloid angioendotheliomatosis are also included in the differential diagnosis [2].

In histopathology, AAD is characterised by a proliferation of small thick-walled vessels (capillaries and/or venules), frequently in a lobular arrangement, and surrounded by pericytes in the papillary dermis [3,6]. Extravasation of erythrocytes and deposition of hemosiderin may be seen [5]. In some cases, plump endothelial cells, an increased number of fibroblasts, dermal fibrosis, papillary dermis oedema, and a superficial mixed inflammatory infiltrate (lymphocytes, histiocytes, and eosinophils) are also observed [2,6].

Because of its clinical resemblance and different prognosis, AAD must be distinguished from KS. KS is a rare angioproliferative neoplasm that primarily affects mucocutaneous tissues and potentially involves internal organs [9]. Although its aetiopathogenesis is incompletely understood, HHV-8 is the major causative agent. It infects the endothelial cell precursors, triggering a hyperinflammatory state and promoting angiogenesis. Immunosuppression and genetic predisposition are also involved [9]. A clinical presumptive diagnosis of KS may be made, but histopathological confirmation is advised, especially in atypical presentations or in association with systemic symptoms [10].

Despite, histopathologic overlap, some features may differentiate the two entities. In KS the vascular proliferation is independent from the underlying vasculature, and the vessels have a jagged appearance [4,5]. In AAD the vascular proliferation results from the preexisting vasculature and the vessels are rounded [4,5]. The growth pattern is also different, with slit-like vascular spaces in KS, instead of a lobular pattern typical of AAD [4,5]. In immunochemistry, perivascular CD34 and nuclear HHV-8 positivity point towards the diagnosis of KS [8]. Additionally, cytologic atypia, common in KS, is absent in AAD [6].

An accurate diagnosis of AAD is essential, as misdiagnosis may lead to inappropriate treatment and a worse prognosis. A major concern is the faulty diagnosis of KS, due to its overall disease-specific mortality rate of 8% [9].

In fact, histopathology plays a main role in the differential diagnosis of AAD. Venous stasis may lead to stasis dermatitis and pigmented purpura, two differential diagnoses of AAD. Stasis dermatitis usually presents with extravasated erythrocytes, hemosiderin-laden macrophages, dermal fibrosis, perivascular lymphocytic infiltration, and proliferation of dilated blood vessels in the papillary dermis [11]. In pigmented purpura, hemosiderin deposition is more superficial [11].

Lichen planus is a lichenoid dermatosis characterised by interface dermatitis, with a band-like lymphocytic infiltrate in the upper dermis obscuring the dermoepidermal junction [12]. Additional findings include acanthotic epidermis with sawtoothed rete ridges, orthohyperkeratosis, wedge shaped hypergranulosis, and a basal vacuolar degeneration with apoptotic keratinocytes (Civatte bodies) [12].

Squamous cell carcinoma and deep mycosis are mandatory differential diagnoses when facing verrucous lesions. Histologically, squamous cell carcinoma is composed by nests of large polyhedral cells, raised nuclear–cytoplasmatic ratios and keratin pearls, sometimes with an overlying dysplastic epithelium [13]. In subcutaneous mycosis, histopathology is an important diagnostic tool. Fungal structures along with granulomatous inflammation, giant cell reaction, and eosinophilic infiltration are usually reported [14]. Fungi can be visualised by special stains, such as Gomori’s methenamine silver (GMS) and periodic acid–Schiff (PAS) [14].

Vascular proliferations, including various subtypes of haemangiomas, should also be considered for differential diagnosis. Spindle cell haemangiomas are well circumscribed dermal nodules, composed of cavernous-like vascular spaces lined by bland endothelial cells, with solid foci of bland spindle cells and epithelioid endothelial cells [13]. Sinusoidal haemangiomas are described as well-circumscribed proliferations of irregular, inter-anastomosing thin-walled vessels, lined by flattened endothelium [13]. Microvenular haemangiomas correspond to proliferations of small dermal venules and capillaries [13]. Angiosarcomas are composed by anastomosing vessels lined by pleomorphic endothelial cells [13]. Lymphangiomas are characterised by thin-walled vascular channels, filed with lymphatic fluid, lined by discontinuous flattened endothelial cells [13].

The treatment of AAD depends on the underlying disease. In chronic venous insufficiency, compression stockings, intermittent pneumatic compression, and limb elevation are the treatment cornerstone [2]. Topical corticosteroids (clobetasol proprionate, 0.05%) and oral antibiotics (erythromycin and dapsone) were also described [5,6]. The mechanism of action of these antibiotics in AAD is unknown. Erythromycin may have anti-inflammatory properties, including the inhibition of leukocyte chemotaxis [7]. In spite of this, oral erythromycin 500 mg four times a day or dapsone 50 mg twice a day for three weeks, have been reported in literature with favourable outcomes [5]. However, the scarce evidence and the uncertain mechanism of action make oral antibiotics an unlikely therapeutic option. A single case report described the successful use of oral propranolol regarding its vasoconstrictive and anti-angiogenic effects [15].

In cases with skin ulceration, local wound care is important [6]. A case report using a tissue matrix–protective agent (heparan sulphate mimetic) was able to heal an ulcerated lesion of AAD of Stewart–Bluefarb type, refractory to other treatment modalities [2].

In arteriovenous malformations or fistulae, obliteration may be achieved by surgery, sclerotherapy, endovenous ablation, and selective embolization [2]. Anticoagulants should be used in thrombophilia and cushions/platforms in amputation stumps [4,16].

Treatment with PDL, although scarcely described in the literature, may be effective in individual lesions [17]. Indeed, PDL should be considered a valid therapeutic option for AAD, given its effectiveness in the treatment of superficial vascular skin lesions. Although PDL is not ablative, it promotes selective photothermolysis of the lesion’s blood vessels without causing direct damage to the epithelium.

In fact, lasers are a well-established option to treat vascular skin lesions, especially for PDL, which has the best efficacy and safety profile. PDL emits a pulsed beam of yellow light at 585 to 600 nm, using a rhodamine dye, dissolved in a solvent, and pumped by a flashlamp [18]. PDL penetrates to a depth of 0.2 mm below the dermoepidermal junction [19]. Larger spot sizes and longer wavelengths allow a deeper penetration that can reach 0.5 to 1.2 mm [19].

The goal of laser treatment is to induce vessel wall damage without affecting perivascular structures. This therapeutic selectivity is achieved by delivering energy precisely into the target based on selective photothermolysis [20]. The energy targets chromophores that, in vascular lesions, is the oxyhaemoglobin present in red blood cells. Its optimal absorption is within 577 to 600 nm, with lasers emitting wavelengths in this range [19]. After absorption, energy is converted to heat and diffuses radially causing a thermal injury. This leads to vascular damage through photocoagulation and/or mechanical injury. When short pulse widths are used, photomechanical damage occurs, causing vessel wall rupture and haemorrhage [18]. Clinically, purpura is visible. On the other hand, with longer pulse-widths, photothermal damage occurs, leading to intravascular coagulation [18]. On physical examination, a subtle darkening of the blood vessel is seen, followed by local erythema and oedema.

Several factors, such as the anatomical site, vessel size and depth, affect laser absorption. Larger and deeper vessels require adjustments in laser technical parameters. A longer wavelength and a larger spot size allow deeper penetration, and a longer pulse width allows treatment of larger-calibre vessels [19]. These adjustments are of particular interest in legs, where vessels tend to be deeper, have larger diameters, thick walls, and contain more deoxyhaemoglobin [18]. In addition, the greater intravascular pressure requires higher fluences.

The common adverse events of PDL include purpura, which appears immediately after the procedure and can last for 7 to 14 days [19]. The occurrence of purpura is related to the pulse duration. Short pulse duration leads to immediate purpura due to vascular rupture and leaking to extravascular tissues [21]. By contrast, long pulse duration minimises the photoacoustic effect to capillary walls [21]. Other adverse events include hyperpigmentation, hypopigmentation, hypertrophic scars, and dermal or epidermal atrophy [19].

Most adults tolerate the pain and discomfort of PDL sessions. However, topical (lidocaine and prilocaine) or local (lidocaine) anaesthesia may be used to reduce these symptoms [19].

In the reported case, PDL selection was based on the histopathological findings. The penetration depth of PDL is more limited than that of Nd:Yag, which reduces its efficacy in treating the underlying vascular malformations. However, considering the reactive vascular proliferation location in the papillary dermis, treatment with PDL was attempted. The described favourable outcomes may be attributed to the efficacy of PDL in treating vascular lesions in the upper dermis.

Finally, partial amputation may be necessary to face serious complications associated with an underlying arteriovenous malformation, such as refractory pain or haemorrhage, recurrent infection, extensive necrotic areas, cardiac insufficiency decompensation, and physical impairment [4]. In the reported case, most of these complications are unlikely, given the characteristics of the venous malformation.

## 4. Conclusions

AAD must be considered in the presence of purple or reddish-brownish papules and plaques on the extremities. Chronic venous insufficiency is the most frequent cause, but investigation of an underlying vascular malformation is essential to prevent complications. Dermatopathology is the gold standard for the differential diagnosis with KS and other conditions such as vascular proliferations, lichen planus, squamous cell carcinoma, deep mycosis, and stasis dermatitis. Management of AAD focuses on treating the underlying cause. Treatment with PDL is scarcely reported in the literature, but it can be useful given the vascular proliferation confined to the papillary dermis. The reported aesthetic improvement and minimal adverse events make treatment with PDL a promising therapeutic option in AAD. In conclusion, this case report highlights histopathology as the cornerstone for differential diagnosis and selection of new treatment modalities.

## Data Availability

The data presented in this study are available on request from the corresponding author due to the case study.
